# Changing Aesthetic Surgery Interest in Men: An 18-Year Analysis

**DOI:** 10.1007/s00266-023-03344-9

**Published:** 2023-05-16

**Authors:** Melinda Lem, Jason T. Pham, Joshua KyungHo Kim, Cathy J. Tang

**Affiliations:** 1 Department of Plastic and Reconstructive Surgery, University of California Irvine School of Medicine, 200 South Manchester Ave., Suite 650, Orange, CA 92868 USA; 2 Division of Plastic and Reconstructive Surgery, Eastern Virginia Medical School, P.O. Box 1980, Norfolk, VA 23501 USA; 3 Duke University School of Medicine, 40 Duke Medicine Circle, 124 Davison Building, Durham, NC 27710 USA

**Keywords:** Aesthetic surgery, Male plastic surgery, Male cosmetic surgery, Nonsurgical procedures, Men, Google trends

## Abstract

**Background:**

Historically, men have been shamed if they cared seemingly too much about their appearance and especially, if they pursued aesthetic surgery. However, due to the changing landscape of the culture, this stigma has seemed to decrease. Men have diverse and quickly changing interests in particular procedures that have not been readily explored in the currently available reports. To examine this, we analyzed interest in specific plastic surgery procedures in men over the last two decades using the Google Trends tool.

**Methods:**

The most common cosmetic procedures were chosen from the American Society of Plastic Surgeons website and served as the search terms for the Google Trends tool from 2004 to 2021. All 19 procedures were examined for overall trends and for changes in the last decade through comparing the data in bisected time periods.

**Results:**

Interest in all plastic surgery procedures in men increased since 2004 except for breast reduction. Most notably, jawline filler, Botox, microneedling, lip filler, chemical peel, CoolSculpting, and butt lift had the largest trend increases. In the last decade, all procedures showed a significant increase in interest.

**Conclusions:**

While surgical volume data are valuable, our study shows that Google Trends is a beneficial tool to predict quickly changing and specific trends, especially as the patient population of plastic surgery grows with increased diversity and generational changes. Our study shows that there is an increase in male-centered plastic surgery procedures, especially nonsurgical facial procedures. Male interest in plastic surgery will continue to increase with time.

**Level of Evidence IV:**

This journal requires that authors assign a level of evidence to each article. For a full description of these Evidence-Based Medicine ratings, please refer to the Table of Contents or the online Instructions to Authors  www.springer.com/00266.

## Introduction

Historically, patients who sought out aesthetic and cosmetic procedures were predominantly women. It has been an assumption that men have long held an unfavorable opinion of aesthetic procedures for the male sex due to stigmatization [1]. However, studies suggest that there is rising body dissatisfaction secondary to increasing use of social media, interactions with peers, and appearance comparisons [2–4]. In addition to this, generational changes, and the increased acceptance of men undergoing cosmetic procedures, the number of men interested in nonsurgical and surgical aesthetic procedures have grown within the last few years, even with discussion in popular magazines and media outlets [5, 6]. In fact, data from the American Society for Aesthetic Plastic Surgeons (ASAPS) showed that there was an approximately 55% increase in plastic surgery procedures in men from 1997 to 2018. [7]

However, because this dataset and other datasets, including the International Society of Aesthetic Plastic Surgery (ISAPS) and American Society of Plastic Surgeons (ASPS), provide the total number of procedures on an annual basis that is released later in the following year and do not provide more specific types of procedures for men, these datasets lack the real-time ability and specificity to determine current interest in those who are curious about the procedure and interested in upcoming plastic surgery trends.

Google Trends is a publicly accessible online platform that allows queries for real-time data within Google Search [8]. It is also capable of reporting frequently searched terms by Google Search users and can be used to combine different search data with other terms to determine associations. For example, studies have used Google Trends to determine a relationship between public interest in plastic surgery after the celebrity popularization of specific procedures [9, 10]. Other studies using Google Trends have shown how plastic surgery had decreased in interest during the COVID-19 pandemic and how it is now experiencing rising interest relative to the pre-COVID-19 period [11–13]

While there are studies that have shown changes in trends of aesthetic surgery procedures, no study has examined the changing interest in aesthetic procedures for men, especially from the start of the 21^st^ century. It is understood that Google Trends may not always translate to surgical volume; however, it is a powerful tool to analyze public interest in particular topics by tracking live search query information where ASPS, ASAPS, and ISAPS data collection are lacking. Therefore, we utilized Google Trends to analyze overall trends in aesthetic nonsurgical and surgical procedures in men from 2004 to 2021 with focus on the last decade.

## Methods

### Data Collection

The most common nonsurgical and surgical procedures were selected from a list of cosmetic procedures published by the ASPS, which resulted in a total of 19 procedures. To each of these procedures, the word “men” or “male” was appended, forming a compound search term. This was done to focus results on searches seeking information about procedures for men. Google Trends was then queried with each compound search term. “Web” searches within the United States were included in this analysis. Hits from “all categories” were counted, with low search volume results removed.

When queried, Google Trends provides the relative search interest for a term. This number represents the popularity of a term, and is calculated by Google Trends during a normalization process. This process starts with the interest in a term being divided by all searches made to Google within a given geographic area and timeframe. This value is then scaled from 0 to 100, depending on all searches for all topics. The resulting value is the relative search interest. These calculations mean that relative search interest for a term cannot necessarily be compared between different regions or timeframes. Instead, it is best used to illustrate trends or how interest has changed over time, not as a direct absolute measurement of how often a term was searched. In obtaining this data, no information about the identity of internet users was gathered or recorded.

Data were collected from January 1, 2004, the earliest date provided, to December 1, 2021, representing an almost 18-year interval. Relative search interest was reported monthly. Overall trends from 2004 to 2021 were reported. The dataset was also bisected into nine-year periods, 2004-2012 and 2013-2021, for analysis of the last decade’s trends.

### Data Analysis

For the overall trends data analysis, the difference in relative search interest was reported, with positive values indicating an increased interest and negative value indicating a decrease in interest.

For the compared datasets, the variance and distribution of the data were determined by Levene’s test and the Shapiro–Wilk test. From these inferential tests, it was determined that a two-tailed Mann–Whitney U test would be most appropriate to determine whether a significant change in relative search interest was observed between the two subsets from the bisected time periods. Significance was evaluated at *p* < 0.05. The effect size was also computed, with values below 0.3 as a small effect size, from 0.3 to 0.5 evaluated as a moderate effect size, and greater than 0.5 effect sizes deemed as large. Analysis was conducted in R version 4.2.0 (The R Foundation for Statistical Computing) in RStudio version using the rstatix and stats packages.

## Results

**Fig. 1:**
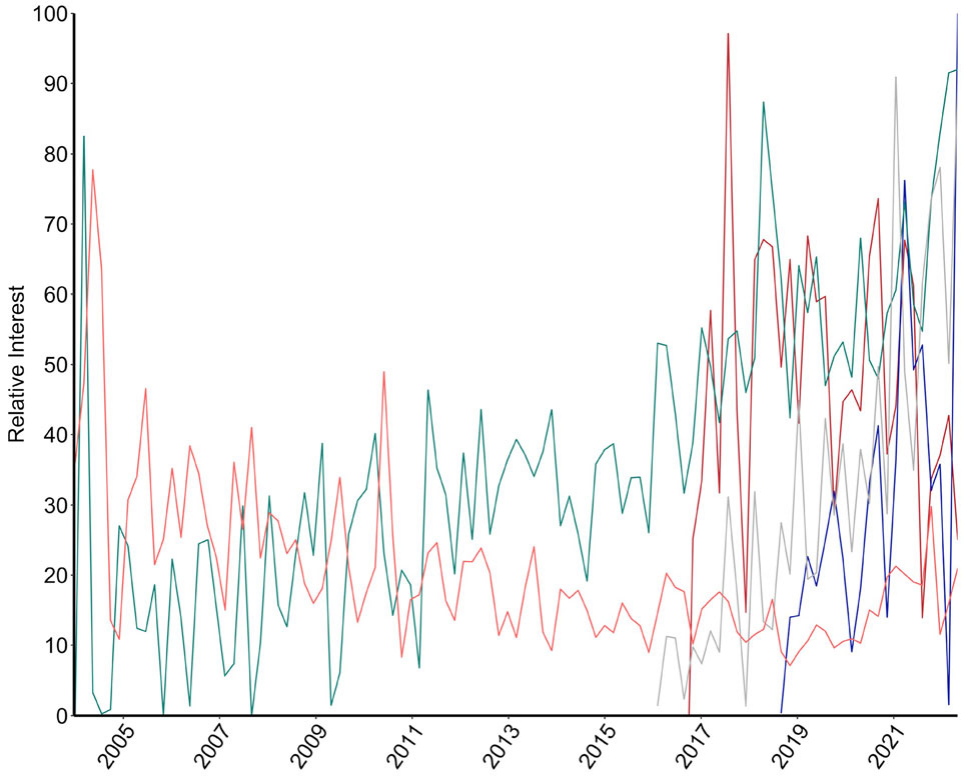
Top 5 procedures in men showing the largest change in interest from 2004-2021**. **Figure Legend: Red: CoolSculpting**,** Orange: Breast Reduction**,** Green: Botox**,** Blue: Jawline Filler**,** Grey: Microneedling

Since 2004, interest in all plastic surgery procedures in men except for breast reduction increased over time (Table 1). The five procedures that had the largest relative search interest increase were jawline filler (70.52), Botox (70.08), microneedling (66.76), CoolSculpting (61.09), and butt lift (60.66) (Table 1 and Fig. 1). Breast reduction had a large decrease in interest over time (− 94.92) (Table 1 and Fig. 1).

**Table 1:** Overall change in interest of plastic surgery procedures in men over 18 years (2004–2021) compared to the American Society of Plastic Surgery (ASPS) surgical volume annual report

Procedure	Change in relative search interest*	ASPS percent change 2020 vs. 2000
Jawline filler	70.52	137%**
Botox	70.08	182%
Microneedling	66.76	–
CoolSculpting	61.09	–
Butt lift	60.66	644%
Chemical peel	59.86	− 79%
Lip filler	55.83	137%
Hair transplant	51.57	− 84%
Tattoo removal	40.27	–
Brazilian butt lift	37.40	–
Neck lift	36.02	–
Microdermabrasion	31.38	13%
Tummy tuck	27.48	63%
Eyelid surgery	24.17	− 21%
Rhinoplasty	23.62	− 57%
Facelift	13.69	19%
Laser hair removal	− 11.69	− 8%
Liposuction	− 31.46	− 56%
Breast reduction	− 94.92	− 9%

*Indexed from 0–100 per procedure in units over time

**ASPS does not specify anatomic location or type of filler, so reported data are all filler data

When looking at the 19 procedures compared between the bisected time periods, three procedures, breast reduction, laser hair removal, and liposuction (all *p* < 0.001), showed significant decreases in relative search interest (Table 2). The remaining 16 procedures had an increase in relative search interest.

**Table 2:** Male plastic surgery interest trends compared between two time periods: 2004–2013 and 2014–2021

Procedure	*p* value	Mean Difs	95% Confidence interval		Effect size	95% Confidence interval	
			Upper	Lower		Upper	Lower
Botox	< 0.001	28.0	33.0	24.0	0.69	0.62	0.76
Brazilian butt lift	< 0.001	12.0	17.0	10.0	0.47	0.34	0.60
Breast reduction	< 0.001	− 9.0	− 7.0	− 11.0	0.58	0.48	0.67
Butt lift	< 0.001	19.0	22.0	17.0	0.56	0.45	0.66
Chemical peel	< 0.001	12.0	14.0	9.0	0.53	0.41	0.65
CoolSculpting	< 0.001	34.0	39.0	28.0	0.80	0.74	0.85
Eyelid surgery	< 0.001	7.0	10.0	6.0	0.33	0.19	0.45
Facelift	< 0.001	10.0	13.0	7.0	0.30	0.16	0.43
Hair transplant	< 0.001	21.0	27.0	15.0	0.43	0.30	0.53
Jawline filler	< 0.001	0.0	0.0	0.0	0.47	0.39	0.55
Laser hair removal	0.01	− 6.0	− 1.0	− 11.0	0.17	0.03	0.30
Lip filler	< 0.001	13.0	17.0	0.0	0.55	0.44	0.65
Liposuction	< 0.001	− 8.0	− 4.0	− 12.0	0.29	0.16	0.42
Microdermabrasion	< 0.001	13.0	15.0	8.0	0.40	0.28	0.53
Microneedling	< 0.001	10.0	18.0	9.0	0.63	0.56	0.71
Neck lift	< 0.001	8.0	11.0	6.0	0.44	0.30	0.56
Rhinoplasty	< 0.001	6.0	9.0	3.0	0.25	0.12	0.37
Tattoo removal	< 0.001	15.0	20.0	10.0	0.32	0.18	0.46
Tummy tuck	< 0.001	9.0	15.0	4.0	0.22	0.09	0.35

All search terms included procedure and “men.” The only procedure that included “male” instead of “men” was breast reduction due to search data availability.

Data was indexed from 0-100 per procedure

Large effect sizes were noted in searches for CoolSculpting (*p* < 0.001, *r* = 0.798), Botox (*p* < 0.001, *r* = 0.694), microneedling (*p* < 0.001, *r* = 0.634), breast reduction (*p* < 0.001, *r* = 0.578), butt lift (*p* < 0.001, *r* = 0.559), lip filler (*p* < 0.001, *r* = 0.547), and chemical peel (*p* < 0.001, *r* = 0.533) (Tables 2 and 3). Breast reduction was the only procedure with a large effect size that displayed a decrease in search interest. For applicable procedures, all procedures except for chemical peel were correlated with matching surgical volume changes from the ASPS 2020 statistics report (Table 1). Moderate effect sizes were observed in eight procedures, most notably Brazilian butt lift (*p* < 0.001, *r* = 0.474), jawline filler (*p* < 0.001, *r* = 0.465), and neck lifts (*p* < 0.001, *r* = 0.439) (Table 2). Small effect sizes were exhibited in four procedures, with the smallest effects seen in laser hair removal (*p* < 0.001, *r* = 0.172) and tummy tuck (*p* < 0.001, *r* = 0.224) (Table 2).

**Table 3:** Top five plastic surgery procedures in men by largest effect size in interest trends

Procedure	*p* value	Mean Difs	95% Confidence interval		Effect size	95% Confidence interval	
			Upper	Lower		Upper	Lower
CoolSculpting	< 0.001	− 34.0	− 39.0	− 28.0	0.80	0.74	0.85
Botox	< 0.001	− 28.0	− 33.0	− 24.0	0.69	0.62	0.76
Microneedling	< 0.001	− 10.0	− 18.0	− 9.0	0.63	0.56	0.71
Breast Reduction	< 0.001	9.0	7.0	11.0	0.58	0.48	0.67
Butt Lift	< 0.001	− 19.0	− 22.0	− 17.0	0.56	0.45	0.66

Data was indexed from 0-100 per procedure

## Discussion

Both surgical volume data and interest data can be valuable to predict what patient and consumer trends will happen in the near future. In the most recent ASPS report from 2020, men had 289,360 aesthetic surgical procedures and 820,123 aesthetic nonsurgical procedures performed [14]. Our study provides interesting context to these reports, showing that men have an increased interest in the majority of major surgical and nonsurgical plastic surgery procedures over the span of almost 20 years, and most notably in the last ten years. This increase in interest can be due to a number of reasons. In a recent survey, almost half of the men surveyed would have a treatment done to “feel better about themselves,” about a quarter of those surveyed men would have a procedure to please their partner, another quarter would like to appear less tired, and another quarter would like to improve their appearance for their career [15]. Additionally, in the broader scope of patients interested in plastic surgery, patients desire an improved appearance, as 79% of facial plastic surgeons report in 2021, compared to 16% reported in 2020 [16]. These reasons may be due to the growing insecurity around appearance due to social media, social comparison, and societal changing and amplified beauty standards [2, 4]. In addition, the changing demographic of millennials as the main plastic surgery consumer market and the increased societal acceptance of men pursuing aesthetic treatments have caused this stigma to change. 31% of surveyed men report to be “extremely likely” to consider a cosmetic procedure, with 92% of these men aged 18-34 years old. [16]

The COVID pandemic also shaped this change in interest. Most patients now have more disposable income due to working from home, as well as more time and flexibility for scheduling and recovery. Additionally, as a consequence of mask-wearing with only the eyes visible, there was a 17% increase in blepharoplasty procedures for more awake-appearing eyes from 2020. [16] However, this increase is not yet reflected in the ASPS data as it contains the full dataset only as recent as 2020. [14]

In the 2020 ASPS report, 2021 The Aesthetic Society report, and 2021 American Academy of Facial Plastic and Reconstructive Surgery (AAFPRS) report, the top cosmetic surgical procedures for men were rhinoplasty, eyelid surgery, cheek implant, breast reduction, liposuction, tummy tuck, and ear surgery [7, 14, 16]. Additionally, instead of multiple facial and body contouring surgeries, male patients are seeking a “Daddy-Do-Over,” which is more common in patients above 40 years old as a parallel procedure to a “Mommy-Makeover” [17]. ASPS data also showed a decrease in gynecomastia/breast reduction procedures over time, which was also reflected in the decreased interest in our analysis.

In these datasets, the top nonsurgical procedures for men included Botox, soft tissue fillers, laser skin resurfacing, laser hair removal, chemical peels, and microdermabrasion [7, 14, 16]. Many of these procedures, most notably Botox, fillers, and microdermabrasion, were largely increased in both interest trends in our study and surgical volume reports over the span of 20 years [14]. Interestingly, the most steeply increasing procedure trends were nonsurgical, such as CoolSculpting, Botox, microneedling, fillers, and chemical peel. Specifically, these were mainly facial nonsurgical procedures, which is consistent with prior studies and the hypothesis that video conferencing, social media, and the focus on the face are increasing awareness of certain aspects of their face and the desire to improve those features. [12, 18]

However, while most interest trends show consistency and predictability with surgical volume, there are a few noted discrepancies. Chemical peel, hair transplant, eyelid surgery, and rhinoplasty surgical volume trends from ASPS 2020 report showed opposite trajectories compared to their interest trends [14]. This could be due to the inclusion of 2021 in the interest trend set while the ASPS procedural volume report only reports up to 2020, thus not showing the full picture. Most notably, it may also be due to the fact that male patients may choose to not include the word “men” or “male” in their search when looking up a procedure, which would be excluded from our analysis.

Limitations in this study include restrictions of Google Trends tool, as the data itself shows search trends and interest, not surgical volume or procedure count. Since the dataset is indexed from 0 to 100, the data numbers do not have an inherent value, especially when used to compare between numbers from other procedures, and only can be analyzed using the slope or data change over time. For example, the trend value of 48 in procedure 1 does not mean that it is more popular than procedure 2 with 30. However, only if multiple procedures are placed into the Google Trends tool and extracted at the same time, the data are normalized and can be compared. This could not be performed due to the maximum of five searches per Google Trends run. Additionally, Google Trends only finds results for specifically searched words, so some trends may have not been properly captured if stated differently than the terms chosen for our study. This dataset also includes all search intentions, such as curiosity about the procedure and market research about the consumers, and thus may not hold the promise of an incoming trend. The changes in interest over time may also be due to the increase in people using Google search and an increase in media, technology, and advertisement overall. This dataset excludes patients who want the procedure, but did not search the procedure on Google, patients who use another search engine, and patients who do not use the internet.

## Conclusion

While surgical volume data are valuable, it is important that plastic surgeons have real-time tools and knowledge to be able to guide their practice, invest in technologies, and predict aesthetic trends. Google Trends is a beneficial tool to predict patient interest and prepare for the future plastic surgery procedural trends, especially as the patient population of plastic surgery grows with increased diversity and generational changes. Our study shows that there is increasing interest from men in the majority of plastic surgery procedures, especially nonsurgical facial procedures such as Botox, fillers, and microdermabrasion over the last 18 years. As shown in both the surgical volume data and our study results, male interest in plastic surgery is only going to continue to increase with time.
